# Dynamic characterization of pathological and functional deterioration in a mouse model of optic neuritis related to neuromyelitis optica spectrum disorder

**DOI:** 10.4103/NRR.NRR-D-24-00898

**Published:** 2025-01-29

**Authors:** Xiayin Yang, Shi-Qi Yao, Henry Ho-Lung Chan, Shaoying Tan

**Affiliations:** 1 School of Optometry, The Hong Kong Polytechnic University, Hong Kong Special Administrative Region, China; 2 Department of Ophthalmology, The First Affiliated Hospital of the Medical College of Shantou University, Shantou, Guangdong Province, China; 3 Research Center for SHARP Vision (RCSV), The Hong Kong Polytechnic University, Kowloon, Hong Kong Special Administrative Region, China; 4 Center for Eye and Vision Research (CEVR), 17W Hong Kong Science Park, Hong Kong Special Administrative Region, China; 5 Research Center for Chinese Medicine Innovation (RCMI), The Hong Kong Polytechnic University, Kowloon, Hong Kong Special Administrative Region, China; 6 University Research Facilities in Behavioral and Systems Neuroscience (UBSN), The Hong Kong Polytechnic University, Hong Kong Special Administrative Region, China

**Keywords:** animal model, aquaporin-4 immunoglobulin G, dynamic profile, electroretinogram, functional deterioration, *in vivo* retinal structural scan, neuromyelitis optica spectrum disorder–related optic neuritis, optic neuritis, pathology, visual-evoked potential

## Abstract

Neuromyelitis optica spectrum disorder–related optic neuritis involves various cellular responses to inflammation and degeneration. In most patients, the primary mechanism underlying neuromyelitis optica spectrum disorder–related optic neuritis is the interaction of aquaporin-4 antibodies with the aquaporin-4 protein present on astrocytes within posterior optic nerve. This binding subsequently initiates a cascade of events leading to secondary demyelination of the optic nerve, ultimately culminating in optic nerve degeneration. Earlier studies on this disorder primarily used systemic-induced animal models, which often require prior activation of a systemic immune response. This can result in primary demyelination of the optic nerve, complicating the interpretation of experimental results. Such methodologies hinder the ability to isolate immune responses triggered by specific antibodies. Additionally, the lack of a detailed profile of disease progression over time limits our capacity to identify potential intervention windows. Therefore, constructing a targeted optic neuritis animal model induced by specific antibodies and elucidate the disease progression arecrucial for exploring the mechanisms underlying neuromyelitis optica spectrum disorder–related optic neuritis. In this study, specific antibodies against aquaporin-4 were precisely injected into the retrobulbar optic nerve of mice to induce a targeted inflammatory response in the posterior optic nerve, resulting in a more representative mouse model of neuromyelitis optica spectrum disorder–related optic neuritis than current models. The progression of the disease was then dynamically observed from both histological and functional perspectives over the course of 1 month following the induction of inflammation. By the first week, astrocytes were damaged, as evidenced by the loss of aquaporin-4 and glial fibrillary acidic protein, the activation of microglia, and the upregulation of microglia-related cytokines, including tumor necrosis factor, interleukin-6, interleukin-1β, C–X–C motif ligand 10, and brain-derived neurotrophic factor. Starting from the second week, there were signs of optic nerve demyelination and significant damage to axonal fibers and retinal ganglion cell bodies. Visual-evoked potentials and dark adaptation threshold responses in electroretinogram both indicated dysfunction in the visual pathway and retina, while optical coherence tomography revealed thinning of the retinal nerve fiber layer in live mice. In summary, in this study we conducted a dynamic exploration of the occurrence and progression of neuromyelitis optica spectrum disorder–related optic neuritis triggered by specific antibodies. Our results show pathological changes at various stages and correlate histological and molecular alterations with *in vivo* structural and functional deterioration. The findings from this study lay an important foundation for further research on neuromyelitis optica spectrum disorder–related optic neuritis.

## Graphical abstract

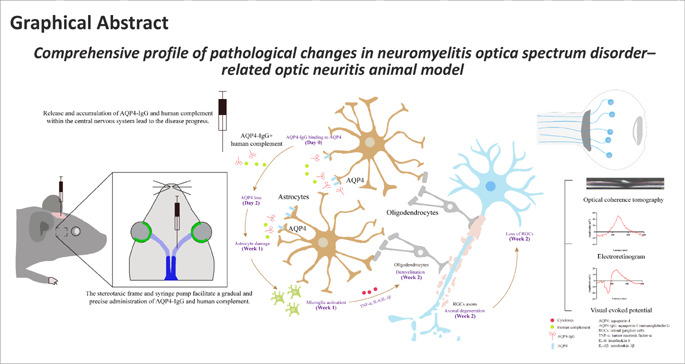

## Introduction

Neuromyelitis optica spectrum disorder (NMOSD) is an autoimmune inflammatory disorder of the central nervous system (CNS) that primarily affects the optic nerves and spinal cord. It is characterized by severe, recurrent episodes of optic neuritis (ON), often resulting in irreversible vision loss and myelitis, and is especially prevalent in Asian populations (Wingerchuk et al., 2015; Tian et al., 2020). NMOSD is frequently linked to pathogenic autoantibodies targeting aquaporin-4 (AQP4), a water channel protein abundantly found on the foot processes of astrocytes, and activation of the complement system, characterized by deposition of complement components such as C5b-9 (Lennon et al., 2005; Wingerchuk et al., 2015; Weinshenker and Wingerchuk, 2017; Asavapanumas et al., 2021). Astrocytes express two isoforms of AQP4, M1 and M23. M23 forms tetramers and orthogonal arrays of particles that bind strongly to AQP4-immunoglobulin G (IgG), while M1 reduces orthogonal arrays of particle size. rAb53 binds more strongly to M23 than rAb58 does, making it more effective at inhibiting AQP4 function (Landis and Reese, 1974; Bennett et al., 2009; Crane and Verkman, 2009; Crane et al., 2011; Wolburg et al., 2011; Phuan et al., 2012; Rossi et al., 2012; Saadoun et al., 2013). AQP4-IgG binding to AQP4 on astrocytes activates the complement system and microglia, which are the primary responders to these signals (Lucchinetti et al., 2014; Chen et al., 2020, 2021; Moinfar and Zamvil, 2020). Cytokines such as interleukin (IL)-6, tumor necrosis factor-α (TNF-α), IL-1β, and C–X–C motif chemokine ligand 10 (CXCL10) influence microglial development and activation. Activated microglia release proinflammatory cytokines, which can harm nearby neurons if activation is chronic (Sui et al., 2006; Wilms et al., 2010; Matsushita et al., 2013; Saadoun et al., 2013; Asavapanumas et al., 2014b; Bradl and Lassmann, 2014; Bennett et al., 2015; West et al., 2019; Fujihara et al., 2020; Yang et al., 2020; Soerensen et al., 2021). In NMOSD-related ON (NMOSD-ON), this inflammation depletes retinal ganglion cells (RGCs) and causes optic nerve degeneration (Asavapanumas et al., 2014b; Bennett et al., 2015; Ransohoff, 2016; Tang and Le, 2016; Oertel et al., 2018; Yang et al., 2020; Oertel et al., 2021; Vegda et al., 2023). In autoimmune diseases, CNS microglia and astrocytes secrete brain-derived neurotrophic factor (BDNF) and nerve growth factor (NGF), which regulate neuroinflammation and neuroprotection, thereby, enhancing neuroplasticity and promoting neuronal survival (De Simone et al., 2007; Rizzi et al., 2018; Tiberi et al., 2022; Nociti and Romozzi, 2023).

The previous investigator attempted to replicate NMOSD-ON through multiple methodologies. The systemic administration of AQP4-IgG in NMOSD-ON may initiate primary demyelination and complicate the ultimate results (Kurosawa et al., 2015; Hillebrand et al., 2019; Remlinger et al., 2023a). Additionally, the localized application of AQP4-IgG to the anterior optic nerve effectively induces NMOSD-ON; however, NMOSD-ON predominantly manifests within the posterior optic nerve (Felix et al., 2016; Zhang et al., 2018; Soerensen et al., 2021; Uzawa et al., 2024). Moreover, previous studies have highlighted the impact of AQP4-IgG on astrocyte damage in the NMOSD-ON animal model. However, the cascading effects on other cell types, such as microglia, oligodendrocytes, and RGCs, remain incompletely understood (Marignier et al., 2016; Sagan et al., 2016). The absence of a detailed timeline for changes like AQP4 loss, astrocyte damage, microglial activation, demyelination, and RGC degeneration in NMOSD-ON hinders the prediction of disease progression and the identification of a disease timeframe (Asavapanumas et al., 2014a, b; Asavapanumas and Verkman, 2014; Bradl and Lassmann, 2014; Zhang et al., 2018; Soerensen et al., 2021).

The aim of this study was to delineate the timeline of disease progression and correlate histological and molecular findings with functional outcomes post-initiation of NMOSD-ON in an optimized animal model. By establishing a comprehensive timeline, we aimed to identify critical windows of disease progression, thereby improving our understanding of NMOSD-ON development.

## Methods

### Ethical approval

This study adhered to the ethical standards of the 1964 *Declaration of Helsinki* and its amendments. The research protocol was approved by The Hong Kong Polytechnic University’s Animal Subjects Ethics Sub-committee (approval No. 20-21/127-SO-R-GRF, approval date: October 7, 2021; approval No. 21-22/152-SO-R-GRF, approval date: February 5, 2024). All animal procedures followed Animal Research: Reporting of *In Vivo* Experiments (ARRIVE) guidelines (Percie du Sert et al., 2020).

### Neuromyelitis optica antibodies

The recombinant monoclonal anti-AQP4 antibody rAb53 (Creative Biolabs Inc., Shirley, NY, USA), also known as AQP4-IgG, was isolated from a clonally expanded population of plasmablasts originating from the cerebrospinal fluid of a patient diagnosed with NMOSD; its derivation and characteristics having been comprehensively documented in earlier studies (Bennett et al., 2009; Crane et al., 2011; Asavapanumas et al., 2014b). Monoclonal recombinant antibodies targeting AQP4 exhibit a greater affinity for antigens than polyclonal antibodies sourced from the sera of NMOSD patients (Duan et al., 2020). Derived from plasmablasts that were clonally expanded from cerebrospinal fluid, recombinant monoclonal NMOSD antibodies were created by co-transfection of heavy and light chain constructs into HEK293 cells. Subsequently, the Fab fragment was obtained through papain digestion of IgG, while the Fc fragment was acquired using protein A (Bennett et al., 2009).

### Establishment of an optimized neuromyelitis optica spectrum disorder–related optic neuritis animal model

All mice were sourced from the centralized animal facilities at the Hong Kong Polytechnic University. Adult female C57BL/6 mice (15–22 g, 8–10 weeks) were housed under a 12/12-hour light/dark cycle with food and water ad libitum, following protocols at the centralized animal facilities at The Hong Kong Polytechnics University. Female mice were selected for our research due to the higher incidence of NMOSD in females. This decision aligns with a prior study (Soerensen et al., 2021), bolstering the reliability of our NMOSD findings. Mice with injured eyes were excluded. All mice were randomly divided into NMOSD-ON and control groups. Initially, five mice underwent euthanasia for tissue sectioning. Following assignment to the groups, five mice each from the NMOSD-ON group and the control group were euthanized on day 2, week 1, week 2, and week 4 for tissue staining. For quantitative reverse transcription polymerase chain reaction (qRT-PCR) analysis, five mice from each group were euthanized at each designated time point. Additionally, at week 4, five mice from each group were euthanized for western blot analysis. Each group comprised 45 mice, resulting in a total of 95 mice, including the five used for baseline analysis. To induce NMOSD-ON, mice were mounted onto a stereotaxic instrument (Portable Stereotaxic Instrument for Mouse, SGL M, Digital. RWD Life Science Co., Ltd., Shenzhen, China) (Asavapanumas et al., 2014b) and subjected to intracranial injection of AQP4-IgG (5 μg, rAb53 variant) and human complement (5 μL, Innovative Research Company, Novi, MI, USA). Animals in the control group were injected with normal IgG (5 μg, Sigma Aldrich Company, Burlington, MA, USA) and human complement (5 μL). The AQP4-IgG/normal IgG and human complement components were combined in a syringe and transferred into a syringe pump (KDS Legato 130 Syringe Pump, RWD Life Science Co., Ltd.). The mice were anesthetized by intraperitoneal injection with a combination of 100 mg/kg ketamine hydrochloride (Alfasan International B.V. Woerden, Holland) and 20 mg/kg xylazine (Alfasan International B.V. Woerden). To perform the intracranial injections, the scalp was thoroughly disinfected, and an incision was made at the top of the mouse skull. A 1-mm aperture was drilled into the skull, positioned 1 mm laterally and 1 mm anterior to the bregma, using coordinates established by the stereotaxic frame. A 33-gauge needle attached to a 25 μL gas-tight syringe was then inserted 6 mm beneath the dura of the skull. This technique guarantees precise placement in the intracranial optic nerve adjacent to the optic chiasm (**Additional Figure 1**). The pump infused the substances slowly into the posterior optic nerve at 1 μL/min, mimicking the gradual buildup of AQP4-IgG and complement activation. Dosage and volume were based on an earlier investigation (Asavapanumas et al., 2014b). Immunoreactive regions were subsequently identified within the intracranial optic nerve (**Additional Figure 1**).

### Experimental timeline

**Figure 1:**
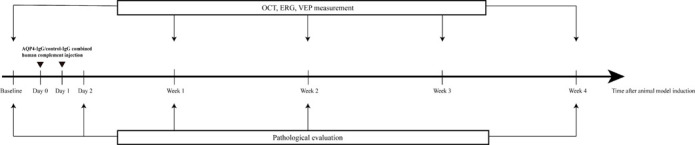
Overview of the experimental timeline. AQP4-IgG: Aquaporin-4 immunoglobulin G; ERG: electroretinogram; VEP: visual evoked potential.

After the initial assessments (baseline) and intracranial injection of AQP4-IgG or normal IgG with human complement, mice in the NMOSD-ON (*n* = 5) and control groups (*n* = 5) underwent further evaluations. Functional changes were assessed at baseline and at weekly intervals (weeks 1–4) using electroretinography (ERG) and visual–evoked potentials (VEPs). *In vivo* structural changes were monitored by optical coherence tomography (OCT) at baseline and weeks 1–4. Histological assessments were conducted at baseline, day 2, week 1, week 2, and week 4 (*n* = 5 per group per time point), as depicted in **Figure 1**.

### Immunostaining for histological assessment

To administer the anesthetic agent, a mixture of ketamine and xylazine was prepared as described above. After anesthesia, a needle was inserted into the left ventricle, and the right atrium was incised. Phosphate-buffered saline was infused into the left ventricle at a rate of 2 mL per minute. Upon completion of perfusion, the entire optic nerve was carefully excised, ensuring that it remained undamaged. Optic nerves and whole-mounted retinas were preserved in 4% paraformaldehyde (Agar Scientific, Sheffield, UK), embedded in O.C.T. compound (SAKURA, Tokyo, Japan), and sectioned into 14-µm slices using a Leica CM1950 cryostat (Wetzlar, Germany). For immunostaining, non-specific binding was blocked with 5% bovine serum albumin in phosphate-buffered saline for 1 hour. The sections were then incubated overnight at 4°C with primary antibodies targeting AQP4 (mouse, 1:200, Santa Cruz, Dallas, TX, USA, Cat# sc-390488, RRID: AB_3083097), glial fibrillary acidic protein (GFAP; rabbit, 1:200, Dako, Glostrup, Denmark, Cat# z0334, RRID: AB_10013382) for astrocytes, CD68 (rat, 1:200, Abcam, Cambridge, UK, Cat# ab53444, RRID: AB_869007) for activated microglia, ionized calcium-binding adapter molecule 1 (Iba-1; rabbit, 1:700, Wako, Osaka, Japan, Cat# 019–19741, RRID: AB_839504) for microglia (Li et al., 2022), myelin oligodendrocyte glycoprotein (MOG; mouse, 1:200, Millipore, Burlington, MA, USA, Cat# MAB5680, RRID: AB_1587278) for myelin (Asavapanumas et al., 2014b), and neurofilament 200 (NF200; mouse, 1:200, Sigma, St. Louis, MO, USA, Cat# N0142, RRID: AB_477257) and brain-specific homeobox/POU domain protein 3A (Brn3a; mouse, 1:200, Millipore, Cat# MAB1585, RRID: AB_94166) for RGC axons and cell bodies (Quina et al., 2005). Subsequently, the sections were washed in phosphate-buffered saline, treated with Alexa Fluor 488 IgG (goat anti rabbit, 1:500, Invitrogen, Waltham, MA, USA, Cat# A11008, RRID: AB_143165), Alexa Fluor 555 (goat anti mouse, 1:500, Invitrogen, Cat#A21422, RRID: AB_141822), Alexa Fluor 488 IgG (donkey anti rabbit, 1:500, Invitrogen, Cat# A21206, RRID: AB_2535792), or Alexa Fluor 555 IgG (donkey anti rat, 1:500, Abcam, Cambridge, UK, Cat# AB150154, RRID: AB_2813834) for 1 hour at room temperature (approximately 25°C), and nuclei were stained with 4′,6-diamidino-2-phenylindole (1:1000, Sigma, D8417) for 15 minutes. Finally, the sections were mounted with anti-fade mounting medium and observed under a fluorescence microscope (Zeiss LSM 800 Upright Confocal Microscope, Carl Zeiss, Oberkochen, Germany). AQP4-IgG and human complement were labeled with and detected using Alexa Fluor 488 IgG (goat anti human-IgG, 1:500, Invitrogen, Cat# A11013, RRID: AB_141360) and anti-C5b9 (mouse, 1:200, Santa Cruz, Dallas, Texas, USA, Cat# sc-66190, RRID: AB_1119840), respectively, at 4°C overnight followed by incubation with the secondary antibody Alexa Fluor 555 (goat anti mouse, 1:500, Invitrogen, Cat#A21422, RRID: AB_141822) at 25°C for 1 hour. Images of cryosections and whole-mounted retinas were taken using a confocal fluorescence microscope with 10×/0.45 and 20×/0.8 objectives. AQP4-IgG and human complement deposition were assessed in 10x confocal images to evaluate both the percentage of the affected area and the immunofluorescence intensity. AQP4, GFAP, MOG, and NF200 immunofluorescence intensity (*n* = 5 for each slide) in 20× confocal images was quantified, using ImageJ software (Version 2, National Institutes of Health, Bethesda, MD, USA), and protein expression was expressed in arbitrary units (Kedziora et al., 2011; Kennedy et al., 2022). Specifically, the confocal microscope settings were standardized, and the images were captured using the same acquisition parameters for one protein expression across all experimental groups and time points (Bonsack et al., 2016; Saraiva et al., 2021). To quantify the number of Brn3a^+^ RGCs cells, three distinct areas in the superior, nasal, inferior, and temporal regions, each consisting 319.5 µm × 319.5 µm squares along the retinal whole-mount axis (20× confocal images), were captured at distances of 200 µm (one image for the central area) and 1 mm (two images for the middle areas) from the optic nerve head, as indicated in **Additional Figure 2**. The number of RGCs was determined using Image J software and averaged for a single sample (entire retina).

### Quantitative reverse transcription polymerase chain reaction analysis of released cytokines

Optic nerves were collected from sacrificed mice at day 2, week 1, week 2, and week 4 post-NMOSD-ON induction after transcardial perfusion with ice-cold phosphate-buffered saline, as described above. Tissues were snap-frozen in liquid nitrogen and stored at –80°C until RNA extraction. Samples were thawed on ice and homogenized in 300 μL of TRIzol (Thermofisher, Waltham, MA, USA) using a mechanical homogenizer. After a 5-minute incubation, 60 μL of chloroform was added, and samples were centrifuged at 12,000 × *g* for 15 minutes at 4°C. The RNA-containing aqueous phase was transferred to a new tube and quantified using a NanoDrop spectrophotometer (Thermofisher), aiming for an absorbance at 260/280 nm ratio of **~**2.0. RNA was reverse transcribed to complementary DNA using a High-Capacity Complementary DN Reverse Transcription Kit (Applied Biosystems^TM^, Thermofisher). qRT-PCR was performed using TB Green Premix Ex Taq II (Takara, Kusatsu, Shiga, Japan) and a QuantStudio^TM^ 7 Flex Real-Time PCR System (Thermofisher). Glyceraldehyde-3-phosphate dehydrogenase (*GAPDH*) was used as the internal control for gene expression analysis (2^–ΔΔCT^). **Table 1** lists all primers utilized in the experimental procedures.

**Table 1:** Primer sequences for the targeted gene

Gene	Forward sequence (5'–3')	Reverse sequence (5'–3')
*GAPDH*	GGG TCC CAG CTT AGG TTC AT	CTC GTG GTT CAC ACC CAT CA
*IL-6*	TAT GAA GTT CCT CTC TGC AAG AGA	CTG CAA GTG CAT CAT CGT TGT TC
*IL-1β*	GGC TGC TTC CAA ACC TTT GA	GAA GAC ACG GAT TCC ATG GT
*TNF-α*	CAG GCG GTG CCT ATG TCT C	CGA TCA CCC CGA AGT TCA GTA G
*CXCL10*	GCC GTC ATT TTC TGC CTC AT	GGC CCG TCA TCG ATA TGG
*BDNF*	TGA CAA CGA CAT CGC ATT AC	TTC AGC CGG TCA GAG AAG
*NGF*	CAA TAG CTG CCC GAG TGA CA	TCC GGT GAG TCC TGT TGA AAG

BDNF: Brain-derived neurotrophic factor; CXCL10: C-X-C motif chemokine ligand 10; GAPDH: glyceraldehyde-3-phosphate dehydrogenase; IL-1β: interleukin 1β; IL-6: interleukin 6; NGF: nerve growth factor; TNF-α: tumor necrosis factor α.

### Western blot assay

All optic nerve tissues obtained from sacrificed mice were denatured by boiling in SDS sample buffer. Subsequently, an aliquot containing 30 µg of total protein was loaded and run on 10% sodium dodecyl-sulfate polyacrylamide gel electrophoresis gels. Following electrophoresis (150 minutes, 300 mA), the proteins were transferred to polyvinylidene fluoride or polyvinylidene difluoride membranes. The membranes were blocked with 5% bovine serum albumin in Tris-buffered saline with Tween® 20 Detergent buffer overnight at 4°C. Then, the membranes were incubated with shaking overnight at 4°C with primary antibodies (anti-NF200, mouse, 1:1000, Sigma, Cat# N0142, RRID: AB_477257 and anti-GAPDH, mouse, 1:1000, Millipore, Cat# CB1001, RRID: AB_2107426). After three washes in Tris-buffered saline with Tween® 20 Detergent, the membranes were incubated with a secondary antibody (goat anti-mouse IgG, conjugated to horseradish peroxidase, ABclonal, Woburn, MA, USA, Cat# AS003, RRID: AB_2769851) diluted 1:1000 in blocking solution at room temperature for 1 hour. The membranes underwent five additional washes before being treated with SuperSignal^TM^ West Pico PLUS Chemiluminescent Substrate (Invitrogen, Waltham, MA, USA) according to the manufacturer’s instructions. The blotting results were analyzed using a ChemiDoc Imager System (BioRad, Hercules, CA, USA). NF200 intensity was normalized to that of the reference protein (GAPDH), which served as a loading control.

### Functional measurement

ERG recordings were conducted to assess retinal function, specifically measuring the positive scotopic threshold response (pSTR), a-wave, and b-wave amplitudes of the scotopic ERG response (Perlman, 1995). Full-field Ganzfeld stimulation (Q450; RETI Animal; Roland Consult, Brandenburg an der Havel, Germany) was used to elicit responses from the inner and outer retinal layers of experimental mice, as described in previous studies (Lakshmanan et al., 2019a, b). Animals were dark-adapted for 12 hours prior to the procedure to ensure sensitivity to scotopic testing. Under dim red light, the mice were anesthetized with a solution consisting of ketamine and xylazine administered via intraperitoneal injection. Pupils were dilated with Mydrin-P (Santen Pharmaceutical, Osaka, Japan). The mice were then placed on a temperature-controlled platform connected to a warm water bath set at 37.8°C. A lubricating gel was applied to prevent corneal dehydration. Recording electrodes (Roland Consult, Brandenburg an der Havel, Germany) were placed on the corneal surface (active electrode), and needles (Roland Consult) were inserted into the lateral canthi of each eye (reference electrode) and the base of the tail (ground electrode). This setup allowed for simultaneous recordings from both eyes. The pSTR was measured by averaging 40 responses with an interstimulus interval of 2 seconds. The stimuli consisted of brief (2 microseconds), dim, white light–emitting diode flashes with intensities ranging from –5.1 to –4.05 log cd·s/m^2^, in steps of 0.15 log cd·s/m^2^. Scotopic (mixed rod and cone) responses were recorded using a single flash with an intensity of 1.3 log cd·s/m^2^. The recorded signals were filtered with a bandpass filter of 0.1 to 1000 Hz. The pSTR amplitudes (at a level around –4.8 log cd·s/m^2^) along with the a- and b-wave amplitudes of scotopic responses were measured and extracted for additional analysis. These responses correspond to the function of RGCs, photoreceptors, and bipolar cells (Perlman, 1995; Saszik et al., 2002), respectively. Full-field ERG was performed to assess retinal function in the NMOSD-ON group and control group at baseline, week 1, week 2, week 3, and week 4.

VEP recordings (non-invasive) were utilized to evaluate the functional integrity of the visual pathway from the retina to the visual cortex (Robson et al., 2018). Animals were anesthetized as described for the ERG recordings, and visual stimuli were presented while the active electrode (a steel needle, (Roland Consult)) was inserted subdermally on the head between the two ears (**Additional Figure 3**). This region corresponds to the area above the cortex where responsive signals are processed. The non-stimulated eye was occluded by a dark patch. The reference electrode (Roland Consult) was placed underneath the tongue (**Additional Figure 3**), while the ground electrode (Roland Consult) was inserted into the tail (Marenna et al., 2019; Liu et al., 2022; Zhang et al., 2024). Full-field (Ganzfeld) stimulation was employed, along with a computation system (RETI port, Roland Consult). The N1-P1 amplitude, N1 latency, and P1 latency of the VEP response were assessed to evaluate the functionality of the visual pathway, as described in previous studies (Zhang et al., 2018; Liu et al., 2022). The N1 wave is the primary negative peak, while the P1 wave is the initial positive peak. N1 latency reflects the timing of the N1 wave and indicates demyelinating injury of the optic nerve (Zhang et al., 2018; Liu et al., 2022). The N1-P1 amplitude was measured from the trough of the N1 wave to the peak of the P1 wave, indicating the integrity of the visual pathway, especially RGCs. P1 latency refers to the time it takes a signal to be transmitted from the retina to the visual cortex. The stimulus and recording parameters aligned with those used in previous studies (Ridder and Nusinowitz, 2006; You et al., 2011; Liu et al., 2022).

### *In vivo* assessment of retinal structure

OCT was conducted using a Bioptigen Envisu spectral domain optical coherence tomography system (Model R2210, Leica Microsystems, Morrisville, NC, USA). OCT was employed to measure the thickness of the retinal nerve fiber layer (RNFL), combined ganglion cell and inner plexiform layer (GCIPL), inner nuclear layer (INL), and inner retina (summing up the RNFL, IPL, and INL), providing indirect *in vivo* assessment of axonal integrity within the retina. The animals were anesthetized, and their pupils were dilated. Rectangular OCT scans were performed (a-/b-scans: 1000 lines; b-scans: 100 scans; frame/b-scans: 10 frames), encompassing an area of 0.8 mm × 0.8 mm. The optic disc was centrally positioned within the scan to serve as a consistent reference point across various subjects and various time points. Retinal thickness was analyzed as previously reported (Lam et al., 2023). The average RNFL, GCIPL, INL, and inner retina thicknesses were calculated to track structural changes in RGCs and axons over time (baseline, week 1, week 2, week 3, and week 4).

### Data collection and analysis

Data for each parameter (immunostaining intensity, ERG, VEP, and OCT) were collected at baseline and in the weeks post-induction. Changes in immunostaining (AQP4, GFAP, MOG, and NF200), microglial counts (Iba-1 and CD68), RGC counts, ERG parameters (pSTR amplitude, a- and b-wave amplitude and latency), VEP parameters (N1-P1 amplitude, N1 and P1 latencies), and OCT measurements (RNFL, IPL, INL thickness) were analyzed using repeated measures. Data are expressed as the mean ± the standard error of the mean (SEM). Comparative analyses were performed using unpaired *t*-tests for two groups and one-way analysis of variance (ANOVA) with Tukey’s test for three or more groups. Two-way ANOVA with *post hoc* Tukey’s correction was used to identify significant temporal variations and group disparities over time. A *P*-value < 0.05 indicated statistical significance. Analyses were conducted using GraphPad Prism (version 9.2.0 for Windows, GraphPad Software, Boston, MA, USA, www.graphpad.com). The first author performed the data analysis blinded to the group assignments. An independent statistician, blinded to the group assignments, also analyzed the data to reduce bias. The sample size was determined based on previous studies (Asavapanumas et al., 2014b; Soerensen et al., 2021; Morita et al., 2022; Remlinger et al., 2023a). Any animals that experienced illness or injury during the course of the experiments were removed from the study. The sample size was then supplemented accordingly to ensure that the statistical integrity of the research was upheld.

## Results

### Temporal progression of pathological changes

#### Early depletion of aquaporin-4 and astrocyte impairment

**Figure 2:**
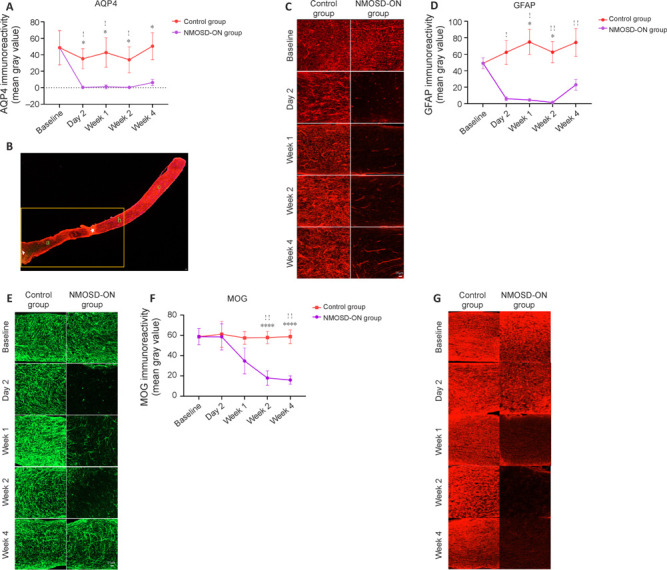
AQP4 protein loss, astrocyte damage (loss of GFAP protein), demyelination (loss of MOG protein), and axonal degeneration (loss of NF200 protein) at different time points. (A) Quantification of AQP4 immunoreactivity (red, Alexa fluor 555) in the optic nerve at different time points, comparing the NMOSD-ON group and the control group or baseline. AQP4 expression exhibited a marked decrease in the NMOSD-ON cohort when compared with both the control group and baseline measurements from day 2 onwards; however, no significant difference was noted at week 4 relative to baseline. (B) Illustration of the lesion involving AQP4 protein loss (orange frame) within the (a) intracranial optic nerve, which corresponds with the injection position, and the (b) posterior optic nerve, but not in the (c) anterior optic nerve. The asterisk marks the intersection of the posterior optic nerve and the intracranial optic nerve, where the optic nerve enters the intracranial cavity. The triangle indicates the region adjacent to the optic chiasm. (C) Representative images of AQP4 loss (red, Alexa fluor 555) in the NMOSD-ON and control groups. (D) Quantification of GFAP immunoreactivity in the optic nerve at different time points, comparing the NMOSD-ON and the control groups or baseline. GFAP expression declined starting from week 2 in the NMOSD-ON group compared with baseline measurements. Additionally, at 1 and 2 weeks there was a significant reduction in GFAP expression relative to the control group. (E) Representative images of GFAP (green, Alexa fluor 488) loss in the NMOSD-ON and control groups. (F) Quantification of MOG immunoreactivity in the optic nerve at different time points, comparing the NMOSD-ON group and the control group or baseline. Notably, from week 2 onwards, there was a marked decrease in MOG expression compared with both the control group and the baseline measurements. (G) Representative images of MOG (red, Alexa fluor 555) loss in the NMOSD-ON group and control group. Scall bars: 20 μm. All data are presented as the mean ± SEM. *P < 0.05, ****P < 0.0001, *vs*. control group; !*P* < 0.05, !!*P* < 0.01, *vs.* baseline (two-way analysis of variance followed by *post hoc* Tukey’s correction). AQP4: Aquaporin-4; AQP4-IgG: anti-aquaporin-4 antibodies; GFAP: glial fibrillary acidic protein; IgG: immunoglobulin G; MOG: myelin oligodendrocytes glycoprotein; NF200: neurofilament 200; NMOSD-ON: neuromyelitis optica spectrum disorders-related optic neuritis.

Temporal changes in AQP4 immunoreactivity in the NMOSD-ON group were assessed at various time points post-induction (day 2, week 1, week 2, and week 4) and compared with the baseline group and the control group. A notable decrease in AQP4 immunoreactivity was observed at the injection site on day 2, indicating an immediate reaction to the presence of AQP4-IgG compared with the control group (*P* = 0.015) and the baseline group (*P* = 0.030). This loss of AQP4 immunoreactivity continued to worsen over time, with reductions at week 1 (*P* = 0.031 *vs*. control and *P* = 0.038 *vs*. baseline), week 2 (*P* = 0.044 *vs*. control and *P* = 0.031 *vs*. baseline), and week 4 (*P* = 0.015 *vs*. control, but not significantly different from baseline) (**Figure 2A–C**).

Concurrently, a decline in GFAP expression was noted, suggesting acute damage to astrocytes. Temporal changes in GFAP immunoreactivity were observed in the NMOSD-ON group at various time points post-induction (day 2, week 1, week 2, and week 4). Quantification of GFAP protein levels revealed significant and progressive loss compared with both the control group and the baseline (pre-induction) group. Briefly, at day 2 post-induction, there was a moderate decrease in GFAP immunoreactivity as compared with the baseline (*P* = 0.0148), which was not significantly different from the control group. However, in week 1 GFAP levels were significantly decreased compared with both the control group (*P* = 0.048) and the baseline group (*P* = 0.016). The decline in GFAP levels persisted at week 2, with a significant decrease compared with the control group (*P* = 0.043) and the baseline group (*P* = 0.008). Interestingly, by week 4 post-induction, there was a slight increase in GFAP immunoreactivity, leading to the absence of a significant difference in GFAP expression between the NMOSD-ON group and the control group. Despite this partial recovery, GFAP levels remained significantly lower at week 4 compared with baseline (*P* = 0.001) (**Figure 2D** and **E**).

To summarize the observed results of astrocytopathy, it indicates that AQP4 protein and astrocytes exhibit degeneration beginning in the early stage of the disease (from day 2 to week 1), yet demonstrate signs of recovery by the late phase of the disease (week 4).

#### Progressive demyelination

The mice in the NMOSD-ON group exhibited a substantial decrease in immunoreactivity for MOG, a key indicator of oligodendrocytes, compared with the control group. From week 2 to week 4 there was a notable reduction in MOG protein levels (immunoreactivity) compared with the control group (*P* < 0.001 and *P* < 0.001 at week 2 and week 4, respectively). Simultaneously, there was a significant decline in MOG protein levels from week 2 to week 4 compared with baseline (*P* = 0.005 and *P* = 0.001 at week 2 and week 4, respectively) (**Figure 2F** and **G**).

By week 2, there is significant initial damage to the myelin sheath, and this demyelination persists until week 4.

#### Activation of microglial and pro-inflammatory cytokines

**Figure 3:**
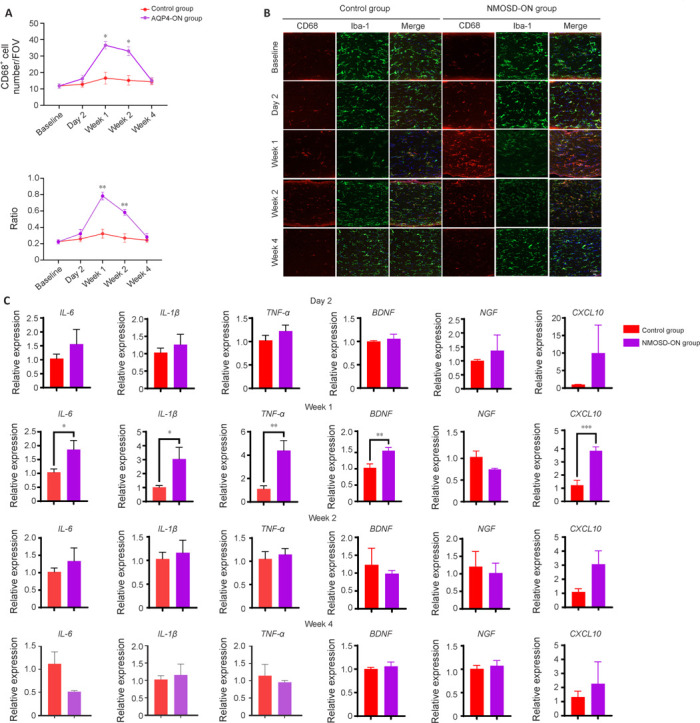
Microglial activation and upregulation of associated cytokines at different time points. (A) Quantification of CD68-labeled microglia in the optic nerve (upper) and temporal changes in the activation status of microglia in the optic nerve, as indicated by the CD68^+^/Iba-1^+^ ratio (lower). (B) Representative images of CD68 (red, Alexa fluor 555) and Iba-1 (green, Alexa fluor 488) immunoreactivity in the optic nerve at different time points, comparing the NMOSD-ON group and the control group. Significant increases in CD68 counts and the CD68^+^/Iba-1^+^ ratio were observed at 1 and 2 weeks. Scall bars: 20 μm. (C) qRT-PCR analysis of the expression of the pro-inflammatory cytokines *IL-6*, *IL-1*β, *TNF-α*, and *CXCL10* and the neurotrophic factors *BDNF* and *NGF* in the optic nerve at 2 days, 1, 2, and 4 weeks, comparing the NMOSD-ON group and the control group. All data are presented as the mean ± SEM. *P < 0.05, **P < 0.01, ***P < 0.001, *vs.* control group (two-way analysis of variance followed by *post hoc* Tukey’s correction for A). The qRT-PCR data in C were analyzed by unpaired *t*-test. BDNF: Brain-derived neurotrophic factor; FOV: field of view; Iba-1: ionized calcium binding adaptor molecule 1; IL-1β: interleukin-1beta; IL-6: interleukin-6; NGF: nerve growth factor; NMOSD-ON: neuromyelitis optica spectrum disorders-related optic neuritis; qRT-PCR: quantitative reverse transcription polymerase chain reaction; TNF-α: tumor necrosis factor-alpha.

The mice in the NMOSD-ON group exhibited a temporal pattern of microglial activation and neuronal damage. CD68 is a marker for activated microglia, although it is not exclusive to this cell type. Iba-1, which is highly expressed by microglia, is a pan-microglial marker that identifies all microglia regardless of activation, while CD68 is linked to microglial phagocytic function. The CD68/Iba-1 ratio indicates the proportion of activated microglia (Ohsawa et al., 2004; Stankov et al., 2015; Wittekindt et al., 2022; Rentsch et al., 2023). CD68^+^ cell numbers significantly increased from week 1 (*P* = 0.012) to week 2 (*P* = 0.011) compared with the control group. The ratio of CD68^+^/Iba-1^+^ cells also significantly increased from week 1 to week 2 (*P* = 0.001 and *P* = 0.009 at week 1 and week 2, respectively, compared with the control group) (**Figure 3A** and **B**).

Next we assessed activation of cytokines in the mouse optic nerve under NMOSD-like conditions by conducting qRT-PCR following the induction of NMOSD-ON to evaluate the gene expression of pro-inflammatory cytokines at week 1, when microglial activation peaks. The results revealed a notable increase in the gene expression levels of *IL-1*β, *IL-6*, *TNF-α*, *CXCL10*, and *BDNF* (*P* = 0.034, *P* = 0.047, *P* = 0.004, *P* < 0.001, and *P* = 0.009, respectively) in the NMOSD-ON group compared with the control group (**Figure 3C**). However, no notable variance in the expression levels of *IL-1*β, *IL-6*, *TNF-α*, *CXCL10*, and *BDNF* was detected at day 2, week 2, and week 4 (**Figure 3C**). Furthermore, no marked increase in *NGF* expression occurred at any of the assessed time points (**Figure 3C**).

Microglial activation is upregulated and peaks at week 1, subsequently diminishing thereafter. Corresponding cytokines also exhibit peak levels at week 1, indicating a maximum state of neuroinflammation during this period.

#### Loss of retinal ganglion cells bodies and axons

**Figure 4:**
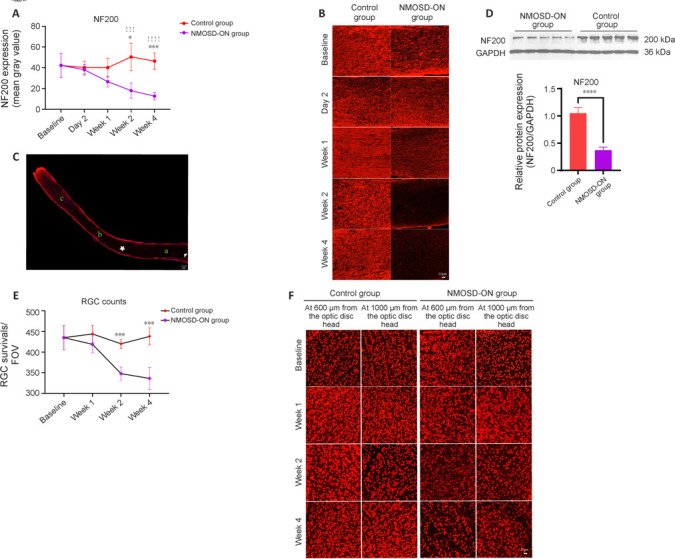
Progressive degeneration of RGC axons and cell bodies in the NMOSD-ON animal model. (A) Quantification of NF200 expression in the optic nerve at different time points, comparing the NMOSD-ON group and the control group or baseline. (B) Representative image of NF200 immunoreactivity (red, Alexa fluor 555) in both the NMOSD-ON group and the control group. At 2 and 4 weeks, NF200 immunoreactivity decreased significantly compared with both the control group and baseline. (C) The whole optic nerve showing widespread NF200 loss involving the (a) intracranial optic nerve, (b) posterior optic nerve, and (c) anterior optic nerve. The asterisk denotes the interface between the posterior optic nerve and the intracranial optic nerve, signifying the location where the optic nerve penetrates the cranial cavity. The triangle designates the area that is adjacent to the optic chiasm. (D) NF200 protein expression, as assessed by western blot (upper), and the comparison of NF200 expression between NMOSD-ON group and the control group (lower). (E) Average number of Brn3a-labeled RGCs in the whole-mounted retina at different time points, comparing the NMOSD-ON group and the control group. (F) Representative images of Brn3a-labeled RGCs (red, Alexa fluor 555). A notable reduction in RGC numbers was observed at 2 and 4 weeks compared with the control group. Scall bars: 20 μm. All data are presented as the mean ± SEM. *P < 0.05, ***P < 0.001, ****P < 0.0001, *vs*. control group; !!!*P* < 0.001, !!!!*P* < 0.0001, *vs*. baseline (two-way analysis of variance followed by *post hoc* Tukey’s correction for A and E). Differences in NF200 expression levels, as detected by western blot, were determined by unpaired *t*-test (D). FOV: Field of view; NF200: neurofilament 200; NMOSD-ON: neuromyelitis optica spectrum disorders–related optic neuritis; RGCs: retinal ganglion cells.

Quantitative analysis of NF200 immunoreactivity, a key indicator of neuronal integrity (Zhou et al., 2021), was conducted at different time points. Our results demonstrated a considerable decline in NF200 immunoreactivity between week 2 and week 4 (*P* = 0.013 at week 2, *P* = 0.001 at week 4 as compared with the control group; *P* < 0.001 at week 2, *P* < 0.001 at week 4 as compared with baseline) (**Figure 4A** and **B**). Axonal degeneration marks the final stage of optic nerve pathology and can result in subsequent retinal involvement. Optic nerve staining revealed widespread loss of NF200 (**Figure 4C**) The decline in NF200 protein expression at week 4, when its reached a minimal level, was validated by western blot analysis (*P* < 0.001 as compared with the control group) (**Figure 4D**).

Moreover, the survival rate (cell numbers) of RGCs in the whole-mounted retina over the course of the study was examined. Our data demonstrated a notable decrease in average RGC numbers in the NMOSD-ON group at week 2 and week 4 compared with the control group (*P* < 0.001 for both time points), indicating a time-dependent depletion of RGCs. No significant differences were observed between the NMOSD-ON group and the control group at baseline or week 1 (**Figure 4E** and **F**).

As a final consequence of NMOSD-ON, optic nerve degeneration, characterized by damage to both axons and neuronal cell bodies, was observed beginning in week 2.

### Evaluation of *in vivo* retinal structure changes by optical coherence tomography

**Figure 5:**
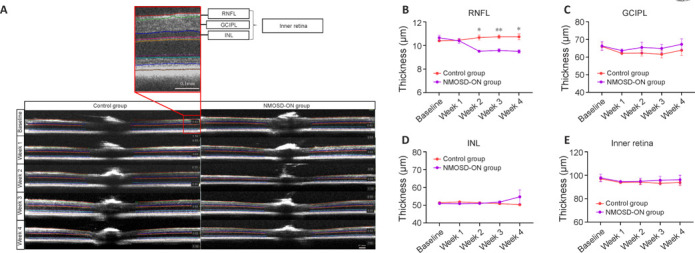
OCT-measured thickness of different retinal layers at different time points in the NMOSD-ON group and the control group. (A) Representative OCT images in the NMOSD-ON group and the control group. The red rectangle frame indicates localization of the RNFL, GCIPL, INL, and inner retina. Scall bars: 0.1 mm. (B) Quantification of RNFL thickness at different time points. (C) Quantification of GCIPL thickness at different time points. (D) Quantification of INL thickness at different time points. (E) Quantification of inner retina thickness at different time points. RNFL thinning became apparent from week 2 onward, but the other layers remained undamaged. All data are presented as the mean ± SEM. *P < 0.05, **P < 0.01, *vs.* control group (two-way analysis of variance with *post hoc* Tukey’s correction). GCIPL: Combined ganglion cell and inner plexiform layer thickness; INL: inner nuclear layer; NMOSD-ON: neuromyelitis optica spectrum disorders-related optic neuritis; OCT: optical coherence tomography; RNFL: retinal nerve fiber layer thickness.

Compared with the control group, the RNFL was significantly thinner in the NMOSD-ON group at week 2, week 3, and week 4 (*P* = 0.018, *P* = 0.002, and *P* = 0.021, respectively) (**Figure 5A** and **B**). No significant differences were observed between the NMOSD-ON group and the control group for the GCIPL, INL, and inner retina at any time point (**Figure 5A** and **C–E**).

The OCT findings corroborated our observations regarding optic nerve degeneration. The RNFL thickness thinning indicated a decline in optic nerve axons beginning at week 2, while the outer retinal layer remained unaffected.

### Assessment of visual pathway impairment by visual–evoked potential and electroretinography

**Figure 6:**
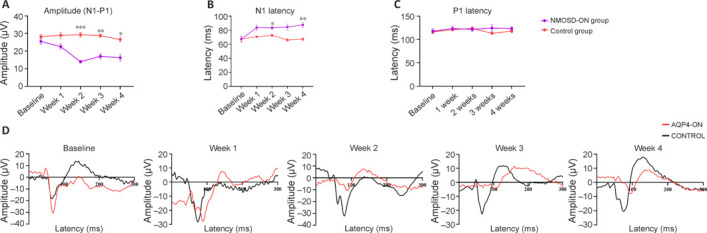
VEP analysis demonstrates progressive functional impairment of the visual pathway in the NMOSD-ON animal model. (A) N1-P1 amplitude of the VEP response in the NMOSD-ON and control groups. (B) VEP N1 latency in the NMOSD-ON and control groups. (C) VEP P1 latency in the NMOSD-ON and control groups. All data are presented as the mean ± SEM. *P < 0.05, **P < 0.01, ***P < 0.001 (two-way analysis of variance with *post hoc* Tukey’s correction). (D) Representative VEP waveforms in both the NMOSD-ON group and the control group. The N1-P1 amplitude reduction and increase in N1 delay became obvious from week 2 onward. NMOSD-ON: Neuromyelitis optica spectrum disorders-related optic neuritis; VEP: visual evoked potential.

**Figure 7:**
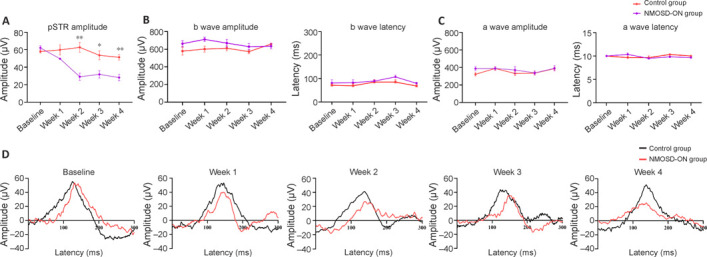
ERG responses reflected functional deterioration of the retina in the NMOSD-ON animal model. (A) ERG pSTR amplitudes in the NMOSD-ON and control groups at different time points. (B, C) Quantification of a- and b-wave amplitude and latency in the NMOSD-ON group and the control group at different time points. All data are presented as the mean ± SEM. *P < 0.05, **P < 0.01 (two-way analysis of variance with *post hoc* Tukey’s correction). (D) Representative images of STR waveforms at different time points in both the NMOSD-ON and control groups. The pSTR amplitude decreased significantly from week 2 onward. ERG: Electroretinogram; NMOSD-ON: neuromyelitis optica spectrum disorders-related optic neuritis; pSTR: positive scotopic threshold response.

The N1P1 wave amplitude in response to VEP, which is indicative of optic nerve functionality, showed a significant decrease in the NMOSD-ON group compared with the control group at week 2 (*P* < 0.001), week 3 (*P* = 0.002), and week 4 (*P* = 0.013) (**Figure 6A** and **D**). Additionally, the NMOSD-ON group showed greater N1 latency at week 2 (*P* = 0.017), week 3 (*P* = 0.052, marginally) and week 4 (*P* = 0.010) than the control group (**Figure 6B** and **D**). However, no significant differences in P1 latency were detected (**Figure 6C** and **D**). ERG revealed progressive inner retinal dysfunction in the NMOSD-ON group. Compared with the control group, the NMOSD-ON group showed a significant decrease in pSTR amplitude at week 2 (*P* < 0.001), week 3 (*P* = 0.002), and week 4 (*P* < 0.001) (**Figure 7A** and **D**). In contrast, no significant differences in either amplitudes or latencies were observed between the NMOSD-ON group and control group for the a- and b-waves of the scotopic ERG response (**Figure 7B–D**).

**Figure 8:**
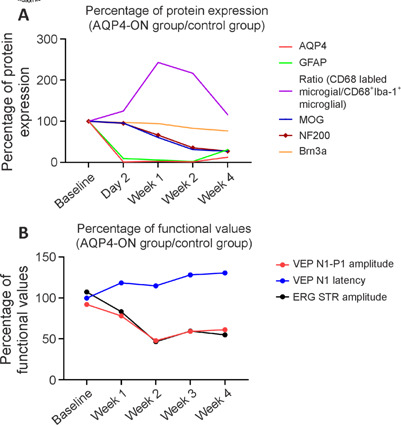
Comprehensive timeline of NMOSD-ON. (A) Protein expression and damage in the NMOSD-ON animal model. The protein expression levels and cell counts in the NMOSD-ON group are presented as a percentage of the control group values (immunofluorescence intensity or cell numbers) over time. (B) Functional evaluation of the NMOSD-ON animal model over time. The functional assessment parameters in the NMOSD-ON group are expressed as a percentage of the control group values over time. AQP4: Aquaporin-4; Brn3a: brain-specific homeobox/POU domain protein 3A; ERG: electroretinography; GFAP: glial fibrillary acidic protein; MOG: myelin oligodendrocyte glycoprotein; NF200: neurofilament 200; NMOSD-ON: neuromyelitis optica spectrum disorders-related optic neuritis; ON: optic neuritis; STR: scotopic threshold response; VEP: visual evoked potential.

Next, the results were summarized as percentages of protein expression and damage levels in the NMOSD-ON group compared with the control group values over time, to observe temporal changes throughout disease progression. AQP4 expression decreased to 1.2% on day 2 and then increased to 12.5% by week 4. GFAP expression decreased to 2.3% at week 2 and then increased to 30.9% by week 4. The proportion of activated microglia increased by 2.43 times and then slightly decreased to 1.16 times by week 4. However, MOG protein expression, NF200 expression, and RGC numbers progressively decreased to 27.1%, 27.5%, and 76.7%, respectively, by week 4. Regarding VEP, the N1-P1 amplitude dropped to 47.7% in week 2 but slightly recovered to 59.1% in week 3 and 61.1% in week 4. The N1 latency progressively increased to 1.18 times in week 1, 1.15 times in week 2, 1.28 times in week 3, and 1.30 times in week 4. Furthermore, the ERG pSTR amplitude decreased to 26.4% in week 2 and then recovered to 59.4% and 54.8% in week 3 and week 4, respectively (**Figure 8A** and **B**).

The identical impairment of inner retinal functionality further corroborates our structural observations. Both the visual pathways and retinal functionality exhibit a decline starting from week 2.

## Discussion

The current study shows a sequence of events: initial AQP4 loss within 2 days, followed by astrocytic damage, microglial activation, demyelination within a week, and RGC neurodegeneration over 2 weeks. For the first time, we assessed retinal and visual pathway functions in an NMOSD-ON model using ERG and VEP. VEP N1 latency analysis identified demyelination alongside MOG protein loss, underscoring the importance of functional evaluations in monitoring NMOSD-ON progression. *In vivo* OCT structural analysis showed that complementary histopathological changes occurred over time.

In this study we established a mouse model of NMOSD-ON. While acknowledging that mice may not fully replicate the complement activation observed in human NMOSD, various studies suggest the value of using mice as a model for studying this disease (Asavapanumas et al., 2014b; Yao et al., 2016; Luo et al., 2020, 2023; Kim et al., 2024). A single injection does not induce NMOSD-ON in an animal model, as it fails to trigger depletion of AQP4 (Asavapanumas et al., 2014b). Using a syringe pump is crucial for replicating the gradual buildup of AQP4-IgG and complement activation seen in patients with NMOSD. In our pilot study, we compared single and double injections (24-hour intervals) of AQP4-IgG to refine our animal model. Double injections led to significantly higher accumulation of AQP4-IgG (*P* < 0.001 as compared with both the control group the and single-injection protocol for both mean grey value and percentage of affected area over total area) and significant recruitment of C5b9 (*P* = 0.030 *vs*. single injection and *P* = 0.005 *vs*. control group) 48 hours post-administration (**Additional Figure 4A–C**). Our pilot study showed that double intracranial injection with a syringe pump efficiently induced autoantibody buildup and complement activation. This highlights the importance of precise AQP4-IgG delivery via repetitive infusion with a syringe pump to establish NMOSD-ON in an animal model (Asavapanumas et al., 2014b). The present model also emphasizes the significance of AQP4-IgG and complement exposure to the intracranial portion of the optic nerve; in contrast, previous NMOSD models have typically targeted the anterior optic nerve (Zhang et al., 2018; Morita et al., 2022), as posterior ON that affects the optic chiasm and the posterior segment of the optic nerve is a notable and common symptom of NMOSD-ON (Caron-Cantin et al., 2019; Wang et al., 2021). This condition can lead to serious and permanent loss of vision (Asavapanumas et al., 2014b; Kim et al., 2015; Srikajon et al., 2018; Zhang et al., 2018; Huda et al., 2019; Prabhat et al., 2021; Sarkar et al., 2021; Wang et al., 2021). Administration of AQP4-IgG should not inflict excessive injury on the mouse. Thus, it is essential to minimize the impact of the injection, as any deviation in the results could jeopardize the integrity of the findings. Osmotic pumps were not used in our study due to the risk of immunological rejection (Asavapanumas et al., 2014b). The smallest possible needle (33 gauge) was used to minimize potential tissue damage. Gradually administering the injection with a syringe pump may also mitigate unexpected pressure-related damage to the blood-brain barrier (Bragin et al., 2018; van den Kerkhof et al., 2024). Accurate stereotactic techniques enhance precision and minimize avoidable tissue injury (Cetin et al., 2006). Three distinct sections were observed in the immunostained optic nerve tissue, consistent with prior research findings (Mesentier-Louro and Liao, 2019). The specific injection site and delivery location were marked by anti-human secondary antibodies and localized within the intracranial optic nerve (**Additional Figure 1**). To ensure consistency, the region of immunoreactivity that was assessed was located in the same area of the intracranial optic nerve across various samples (**Additional Figure 1**).

The observed loss of AQP4 protein at day 2 in our model corresponds with the pathological characteristics of NMOSD in human autopsy samples (Roemer et al., 2007; Lucchinetti et al., 2014). Similar findings have been reported in other NMOSD animal models (Phuan et al., 2012; Morita et al., 2022; Li et al., 2024; Xu et al., 2025). The reported timing of AQP4 loss varies in the literature and may be influenced by the administration route. Direct approaches, like intracranial injection (Ratelade et al., 2013; Bonsack et al., 2016), intrathecal injection (Soerensen et al., 2021), and injection underneath the optic nerve sheath (Zhang et al., 2018; Morita et al., 2022) could lead to quicker AQP4 loss, demyelination, and axonal loss manifestation compared with systemic administration (Bennett et al., 2009; Bradl et al., 2009; Kinoshita et al., 2009). Engineered AQP4-IgG offers improved binding affinity and specificity over the diverse antibodies present in patient-derived AQP4-IgG (Phuan et al., 2012; Tradtrantip et al., 2012). The homogeneity of the recombinant antibodies can lead to more consistent and efficient activation of the complement system, speeding up AQP4 loss (Lennon et al., 2005; Phuan et al., 2012). The early reduction in AQP4 expression in NMOSD suggests that optic nerve involvement begins earlier than previously thought, altering the disease timeline (Duan and Verkman, 2020; Soerensen et al., 2021). This highlights AQP4 loss as a key factor in initiating inflammation in NMOSD-ON. Further research into early molecular events could uncover new biomarkers and insights into disease progression.

We observed temporal fluctuations in GFAP immunoreactivity in our NMOSD-ON animal model. The observed decrease in GFAP expression in week 1, followed by a partial recovery at week 4, aligns with the established astrocyte pathology in NMOSD (Saadoun et al., 2010; Hinson et al., 2012; Phuan et al., 2012; Asavapanumas et al., 2014b; Lucchinetti et al., 2014; Zhang et al., 2018). The reduction suggests astrocyte injury peaks in week 1, a critical time for therapy to prevent irreversible damage. Partial GFAP recovery by week 4 hints at astrocytic resilience or regeneration, though full recovery seems unlikely. Understanding natural repair processes is vital for clarifying disease progression and recovery, and could potentially reveal key biological mechanisms essential for recovery (Lucchinetti et al., 2014; Zhang et al., 2018; Guo et al., 2022).

In our study, microglial upregulation was not immediately apparent at day 2 but became pronounced by week 1 post-induction. Interestingly, this heightened microglial activation was not maintained; instead, a gradual decrease in microglial activity was noted in weeks 2 and 4. This reduction in microglial activity may suggest resolution of the inflammatory response (Sinner et al., 2023; Luo et al., 2025). Early microglial activation may remove damaged cells, but can also worsen neuronal injury by increasing the release of pro-inflammatory cytokines (e.g., TNF-α, IL-1β, IL-6, CXCL10) (Sui et al., 2006; Marín-Teva et al., 2011; Chen et al., 2020; Li et al., 2021). In the NMOSD-ON model, increased cytotoxic cytokine and BDNF levels in optic nerve tissue at week 1 suggests that peak inflammation occurs early (Lee et al., 2002; Cheeran et al., 2003; Wilms et al., 2010; West et al., 2019; Nociti and Romozzi, 2023). The decline in microglial activation by week 4 indicates a shift from acute to chronic inflammation in the optic nerve, signaling CNS recovery. Peak activation marks a critical disease stage, while decreased activation suggests progression toward repair. Monitoring these changes is essential for understanding disease phases, with post-peak activation indicating a transition to restoration. Biomarker changes can reveal disease progression and resolution (Yamasaki et al., 2014; Chen et al., 2020, 2021).

A marked decline in the levels of MOG, an oligodendrocyte marker, in the NMOSD-ON animal model signals demyelination (Tea et al., 2019). Oligodendrocytes are crucial for the maintenance of myelin, which is vital for CNS electrical conduction (Reindl and Waters, 2019). In contrast to multiple sclerosis, NMOSD demyelination stems from astrocyte damage and inflammation (Marignier et al., 2010; Wrzos et al., 2014). These findings align with NMOSD-ON pathology in humans, which is characterized by autoimmune oligodendrocyte destruction (Lucchinetti et al., 2014; Zhang et al., 2018; Tea et al., 2019). The notable drop in MOG levels from week 2 to week 4 relative to baseline indicates ongoing oligodendrocyte destruction in the NMOSD-ON model (Wrzos et al., 2014).

The observed reduction in NF200 expression and the decrease in RGC counts in our NMOSD-ON model align with retinal involvement in NMOSD-ON, which is characterized by axonal damage and RGC loss (Asavapanumas et al., 2014b; Lucchinetti et al., 2014; Duan and Verkman, 2020; Carnero Contentti and Correale, 2021). These changes follow the clinical progression of NMOSD, where ON leads to visual impairment (Lucchinetti et al., 2014; Finke et al., 2018; Wu et al., 2019; Carnero Contentti and Correale, 2021). The absence of significant differences at day 2 and week 1 suggests that early disease stages may not involve overt neuronal damage, consistent with studies indicating delayed neurodegeneration after inflammatory attacks (Asavapanumas et al., 2014b; Zhang et al., 2018).

Loss of MOG, NF200, and RGC cell bodies by week 4 indicates significant, possibly irreversible, damage affecting both myelin and neurons. Investigating late NMOSD-ON stages is crucial for understanding myelin and axonal injury. An integrated approach targeting inflammation, myelin repair, and neuroprotection is essential.

The progressive RNFL thinning observed in OCT scans of mice in the NMOSD-ON group aligns with findings from patients with NMOSD-ON (Ratchford et al., 2009; Costello, 2011; Monteiro et al., 2012; Bennett et al., 2015; Wingerchuk et al., 2015). Significant RNFL changes emerging as early as 2 weeks correspond with the RGC degeneration seen in previous models (Asavapanumas et al., 2014b; Duan and Verkman, 2020), suggesting RNFL loss as a structural biomarker for NMOSD-ON retinal pathology. Interestingly, there were no notable differences in the inner retinal layers (INL and GCIPL) between the NMOSD-ON and control groups, reflecting the selective RNFL vulnerability seen in human studies (Ratchford et al., 2009; Mateo et al., 2017; Oertel et al., 2021). Our multimodal approach, combining OCT and histology, underscores OCT’s value as a non-invasive tool for monitoring NMOSD-ON progression.

ERG and VEP tests revealed functional impairment linked to NMOSD-ON pathology, underscoring their role in evaluating disease progression and treatment efficacy (Wrzos et al., 2014). The selective deficit in seen in pSTR during ERG tests, with stable a- and b-waves, suggests that inner retinal neurons, especially RGCs, are primarily impacted. An innovative aspect of this study is that we examined retinal function in an NMOSD-ON animal model using electrophysiological metrics to evaluate retinal integrity (Alarcón-Martínez et al., 2009, 2010; Pérez de Lara et al., 2014; Lakshmanan et al., 2019a, 2023). The pattern of pSTR amplitude decline correlated with structural and functional deficits noted in OCT and VEP studies, providing evidence of progressive inner retinal pathology. The absence of significant delays in scotopic ERG a- and b-waves might result from secondary demyelination occurring post-astrocyte damage in NMOSD-ON, in contrast with primary myelin sheath injury in MS-ON (Lucchinetti et al., 2014; Hanson et al., 2022). A previous study (Zhang et al., 2018) reported a marked decline in VEP amplitude within the first week, and our findings are consistent with this. The alterations in N1 latency seen in our study correspond with pathological demyelination, establishing its significance as a marker for demyelination and potential utility for *in vivo* monitoring (You et al., 2011). This monitoring is vital for implementing timely interventions that enhance remyelination and slow disease progression (You et al., 2011; Franklin and Ffrench-Constant, 2017; Zheng et al., 2022).

Systemic induction of NMOSD-ON in animals reflects that NMOSD is a systemic disorder, provides a holistic understanding of the disease, and can be used to simulate blood–brain barrier disruption through pre-existing experimental autoimmune encephalomyelitis (Bennett et al., 2009; Constantinescu et al., 2011; Duan and Verkman, 2020; Remlinger et al., 2023b). However, as experimental autoimmune encephalomyelitis might play a role in demyelination, it can complicate detection of AQP4-IgG’s specific effects (Bennett et al., 2009; Duan and Verkman, 2020; Liu et al., 2024). The current study’s approach specifically highlights the effects of AQP4-IgG, capturing the sequential processes of AQP4 loss, astrocyte injury, and optic nerve demyelination. By isolating AQP4-IgG’s effects, this method allows for targeted investigation of NMOSD-ON’s pathogenic mechanisms, free from experimental autoimmune encephalomyelitis-related confounding factors. This specificity provides a clearer understanding of AQP4-IgG’s direct impact on disease progression and optic nerve pathology. Additionally, systemically-induced NMOSD-ON does not effectively replicate the retinal involvement seen in human NMOSD-ON (Saini et al., 2013; Zeka et al., 2015; Sagan et al., 2016). The successful induction of retinal involvement in our NMOSD-ON model via direct AQP4-IgG delivery to the optic nerve enables the study of progressive retinal histological and functional changes. Additionally, using female animals maintains consistency with prior research and reflects the sex disparity seen in human patients (Huda et al., 2019).

The primary limitation of our study is the cross-sectional design used for histological evaluation, which may have missed elements of the dynamic progression of NMOSD-ON pathology. This snapshot approach could have overlooked crucial transitional stages or variations in disease progression. While OCT measures retinal layer thickness *in vivo*, it does not directly observe cellular bodies. Additionally, the study did not explore interactions among different proteins and cell types associated with NMOSD-ON. Future research should investigate these inter-protein and intercellular relationships for a more comprehensive understanding of the disease. Furthermore, the observation period could be extended beyond 4 weeks to assess the potential for auto-recovery from NMOSD-ON. However, numerous studies show that significant changes and injury in NMOSD often occur in the first four weeks after induction in animal models. Our timeline aligns with these established norms (Asavapanumas et al., 2014b; Zhang et al., 2018; Soerensen et al., 2021; Morita et al., 2022). Our main research questions focused on the gradual pathological deterioration seen in NMOSD-ON, where injury persists and cannot be reversed without proper treatment (Carnero Contentti and Correale, 2021; Fadda et al., 2022); for this reason, we believed a 4-week timeframe to be appropriate. In future investigations, we will prioritize prolonged observation periods to explore the critical pathological changes associated with long-term impact and recovery.

In summary, the detailed characterization of pathological changes—from initial AQP4 reduction to astrocyte damage, microglial activation, demyelination, and neurodegeneration—provides a comprehensive profile of our NMOSD-ON animal model. The VEP and ERG findings underscore the utility of functional readouts for monitoring demyelination and axonal degeneration. RNFL thinning, as assessed by OCT, serves as a precise biomarker for disease progression.

## Additional files:

***Additional Figure 1:*** *Confirmation of successful delivered AQP4-IgG and human complement.*

Additional Figure 1 Confirmation of successful delivery of AQP4-IgG and human complement.
Deposition of AQP4-IgG (green, stained with anti-human IgG, Alexa fluor 488), indicating delivery to the (a) intracranial optic nerve but not the (b) posterior optic nerve or (c) anterior optic nerve. The asterisk indicates the junction between the posterior optic nerve and the intracranial optic nerve, marking the point at which the optic nerve enters the cranial cavity. The orange frame indicates the area evaluated for quantification of immunoreactivity. The red triangle is adjacent to the optic chiasm. AQP4-IgG: Anti-aquaporin-4 antibodies.

***Additional Figure 2:*** *Sampling regions for RGCs counts.*

Additional Figure 2 Sampling regions for RGC counts.
Brn3a^+^ RGC cells were counted within three separate 319.5 μm × 319.5μm sampling regions (one central region at a distance of 200 μm and two middle regions at distances of 1000 μm from the optic nerve head) within the superior, nasal, inferior, and temporal retinal quadrants. Brn3a: Brain-specific homeobox/POU domain protein 3A; RGCs: retinal ganglion cells.

***Additional Figure 3:*** *Electrodes installation for VEP.*

Additional Figure 3 Electrode insertion for VEP.
The active electrode, a steel needle, was inserted subdermally in the area between the ears (corresponding to the cortex), while the reference electrode was placed underneath the tongue. VEP: Visual evoked potential.

***Additional Figure 4:*** *Passive transfer of AQP4-IgG (double injections) results in substantial accumulation of AQP4-IgG and human complement.*

Additional Figure 4 Passive transfer of AQP4-IgG (double injections) results in substantial accumulation of AQP4-IgG and human complement.
(A) Quantification of AQP4-IgG immunoreactivity (green, Alexa fluor 488) after a single injection or double injection with a 24-hour interval. The left panel of the figure depicts the mean grey value of AQP4-IgG in the posterior optic nerve, while the right panel illustrates the percentage of AQP4-IgG deposition. (B) Quantification of C5b9 deposition (red, Alexa fluor 555) in the optic nerve at day 2 after a single injection or double injection with AQP4-IgG. Left: C5b9 immunoreactivity after double injection compared with single injection and the control group; right: the affected area. All data are presented as the mean ± SEM. ^*^P < 0.05, ^**^P < 0.01, ^***^P < 0.001, ^****^P < 0.0001 (one-way analysis of variance with Tukey’s test) (C) Representative image showing accumulation of AQP4-IgG and C5b9 under the indicated conditions (double injection, single injection, and control group). Scale bar: 20 μm. AQP4: Aquaporin-4; ERG: electroretinogram; GFAP: glial fibrillary acidic protein; IgG: immunoglobulin G; OCT: Optical coherence tomography; VEP: visual evoked potential.

## Data Availability

*All data relevant to the study are included in the article or uploaded as Additional files.*
